# Socially proximate firearm violence across the life course: a population-based study of California adults

**DOI:** 10.1186/s40621-026-00704-2

**Published:** 2026-08-11

**Authors:** Nicole Kravitz-Wirtz, Amanda J. Aubel

**Affiliations:** Department of Emergency Medicine, Centers for Violence Prevention, University of California Davis School of Medicine, 4301 X St, Sacramento, 95817 CA USA

## Abstract

**Background:**

The effects of firearm violence extend beyond those who are directly injured, affecting families, friends, and communities through experiences such as knowing someone who has been shot. Although indirect exposure to firearm violence has been associated with adverse health outcomes, less is known about the prevalence, timing, and cumulative burden of socially proximate firearm violence exposure across the life course.

**Findings:**

We analyzed data from the 2024 California Safety and Wellbeing Survey, a probability-based survey of California adults (*N* = 3,531). Overall, 20.7% (95% confidence interval [CI] = 18.8–22.8) reported personally knowing someone who had been intentionally shot, representing an estimated 6.3 million California adults. Median age at first exposure was 19 years (interquartile range = 13–27), and more than half of exposed respondents reported knowing multiple people who had been shot. By age 45, cumulative exposure reached 51.3% (95% CI: 40.9–63.2) among Black individuals, 22.9% (95% CI = 19.2–26.6) among Latino/a/x individuals, and 17.9% (95% CI = 15.1–20.8) among White individuals. Compared with White individuals, Black (hazard ratio = 3.85, 95% CI = 2.64–5.62) and Latino/a/x (hazard ratio = 1.42, 95% CI = 1.10–1.83) individuals experienced significantly higher hazards of exposure.

**Conclusions:**

Socially proximate firearm violence is a common life-course experience that often begins during adolescence. These findings provide the first population-based estimates of its timing and cumulative burden, establish a foundation for surveillance and future research on the population health consequences of broader experiences of firearm violence, and can inform developmentally and culturally responsive early intervention and trauma recovery efforts.

**Supplementary Information:**

The online version contains supplementary material available at https://doi.org/10.1186/s40621-026-00704-2.

## Introduction

Firearm violence produces harms that extend beyond those who experience victimization directly [1]. This broader conceptualization of firearm violence exposure aligns with scholarship on “secondary victims” and “homicide survivors,” which emphasizes that violence-related loss reverberates through families, peer groups, and communities, producing trauma, grief, and social disruption among surviving loved ones and community members [2]. Black Americans and other marginalized racial and ethnic groups shoulder a disproportionate share of this burden due to systemic inequities that shape exposure to homicide, the distribution of its downstream consequences, and access to culturally responsive resources and supports within impacted communities [3, 4, 5, 6].

From this perspective, firearm violence constitutes not only an acute event affecting primary victims, but also a collective phenomenon with population health and health equity implications [7]. Indirect exposure to firearm violence has been associated with elevated posttraumatic stress symptoms, depression, anxiety, substance use disorders, and, more recently, poorer physical wellbeing [8, 9, 10, 11]. These impacts extend to the social networks of individuals affected by both fatal and nonfatal shootings [12, 13]. National surveys have documented widespread indirect exposure to firearm violence [14, 15, 16], with the most recent national survey estimating that approximately one in five U.S. adults know a family member or friend who has been intentionally shot by another person [16]. However, these studies have primarily estimated lifetime prevalence and have not characterized when exposure first occurs or how it accumulates across the life course. This gap is particularly important given that indirect firearm violence exposure may function as a significant traumatic stressor during early and other formative stages of development [17, 18].

This short report therefore examines cumulative experiences of socially proximate firearm violence exposure through midlife, defined here as personally knowing someone who has been shot by another person on purpose, using statewide survey data from California. To our knowledge, this is the first general population study to characterize the distribution of these experiences across the life course. We additionally examine inequities in cumulative exposure and the timing of first exposure across racial and ethnic subgroups. Findings may help inform more developmentally appropriate and equity-oriented approaches to prevention, trauma recovery services, and community healing.

## Methods

Data are from the California Safety and Wellbeing Survey (CSaWS), a repeated cross-sectional statewide internet survey examining violence-related topics. The survey was fielded from 4 to 25 November 2024 using probability-based sampling from the Ipsos KnowledgePanel to recruit 5,841 participants ≥ 18 years residing in California households, of whom 3,631 (62.2%) completed the survey. The analytic sample included 3,531 respondents after excluding those who refused more than half of survey items (*n* = 13) or provided an invalid ZIP code (*n* = 87).

The University of California, Davis Institutional Review Board approved all procedures and issued an exempt determination; participants provided electronic informed consent, and the research team did not have access to directly identifying information.

### Measures

Respondents were asked whether someone they personally knew had ever been “shot by another person on purpose,” excluding law enforcement and military personnel injured in the line of duty. Those reporting exposure were additionally asked how many people they knew who had been shot, their age at first exposure, and their relationship to the first person shot.

Race and ethnicity were jointly categorized using separate questions assessing Hispanic or Latino ethnicity and race. Respondents identifying as American Indian or Alaska Native, Asian, Native Hawaiian or other Pacific Islander, or more than one race were combined into an “other/multiracial” category because of small sample sizes.

### Statistical analysis

Analyses were conducted in Stata 15.1 (StataCorp) using post-stratification survey weights to adjust for selection probability and nonresponse and generate estimates representative of California adults based on the 2018–2022 American Community Survey.

Standard descriptive statistics (weighted prevalence estimates and 95% confidence intervals [CIs]) were calculated. Kaplan-Meier survival methods were used to estimate cumulative exposure to socially proximate firearm violence through age 45, overall and by race and ethnicity. Age at first exposure was modeled as a time-to-event outcome, with unexposed respondents censored at their age at survey completion. Because variance estimation for weighted Kaplan-Meier estimates was unavailable, 95% CIs were derived from 500 bootstrap replications. Cox proportional hazards models were used to examine associations between race and ethnicity and exposure, with results reported as hazard ratios (HRs) and 95% CIs.

## Results

Overall, 20.7% (95% CI = 18.8–22.8) of California adults reported ever personally knowing someone who had been shot (Table 1). More than half of exposed individuals reported knowing multiple people, including 5.5% (95% CI = 4.5–6.7) who knew two people and 5.9% (95% CI = 4.8–7.3) who knew three or more people who had been shot. Exposure was especially concentrated among Black individuals, 30.5% (95% CI = 20.9–42.2) of whom reported knowing three or more people who had been shot. Among those with lifetime exposure, 9.6% (95% CI = 6.6–13.7) reported knowing someone who had been shot within the past 12 months. First exposure typically occurred during adolescence and early adulthood (median age = 19 years, IQR = 13–27), occurring progressively earlier among individuals with greater lifetime exposure, including a median age of 14 years (IQR = 10–21) among those who knew three or more people who had been shot. The first person known to have been shot was most often described as a friend or extended family member, although reports of a child, parent, or spouse were identified relatively more frequently by Black and Latino/a/x individuals than others.

**Table 1:** Prevalence estimates of lifetime exposure to socially proximate firearm violence among California adults, overall and by race and ethnicity, 2024 California Safety and Wellbeing Survey

	Overall	White	Black	Latino/a/x	Other/Multiracial
***Among full sample (N = 3531):***					
**Race and ethnicity**	-	39.7 (37.3–42.1) [1783]	5.2 (4.2–6.5) [215]	35.9 (33.5–38.4) [1118]	19.2 (17.1–21.5) [415]
Lifetime exposure to socially proximate firearm violence*					
Yes, knows 1 + person who was shot	20.7 (18.8–22.8) [782]	18.8 (16.2–21.8) [339]	51.6 (40.5–62.6) [104]	23.0 (19.7–26.8) [279]	11.7 (8.3–16.4) [60]
No, does not know anyone who was shot	78.6 (76.5–80.6) [2732]	80.4 (77.3–83.1) [1436]	47.7 (36.8–58.9) [110]	76.2 (72.3–79.6) [833]	88.1 (83.5–91.6) [353]
**Frequency of exposure in lifetime**					
Knows 1 person who was shot	8.7 (7.4–10.1) [345]	10.4 (8.5–12.8) [196]	9.0 (5.2–15.2) [22]	8.3 (6.2–11.0) [100]	5.6 (3.3–9.3) [27]
Knows 2 people who were shot	5.5 (4.5–6.7) [222]	5.5 (3.9–7.7) [87]	11.7 (6.4–20.2) [25]	6.7 (5.0–9.0) [94]	1.7 (0.9-3.0) [16]
Knows 3 + people who were shot	5.9 (4.8–7.3) [200]	2.6 (1.8–3.7) [52]	30.5 (20.9–42.2) [55]	7.2 (5.2–9.8) [78]	3.9 (2.0-7.4) [15]
***Among those with lifetime exposure (N = 782)***:					
**Exposure during past 12 months**	9.6 (6.6–13.7) [63]	4.6 (1.6–12.0) [8]	16.7 (7.1–34.5) [13]	13.2 (8.3–20.4) [36]	4.5 (1.8–10.7) [6]
**Median age at first exposure (IQR)**,** years**	19 (13–27)	22 (16–32)	16 (10–22)	16 (11–25)	18 (14–25)
Knows 1 person who was shot	22 (16–32)	25 (18–35)	22 (10–45)	20 (15–30)	19 (18–27)
Knows 2 people who were shot	19 (14–25)	20 (16–25)	18 (13–21)	17 (13–21)	25 (19–29)
Knows 3 + people who were shot	14 (10–21)	17 (14–25)	13 (10–21)	12 (8–20)	14 (14–21)
**Relationship of first person shot to respondent**					
Friend	35.0 (30.1–40.3) [273]	37.7 (30.1–46.0) [130]	37.6 (23.4–54.2) [32]	27.7 (21.0-35.6) [85]	49.9 (32.6–67.2) [26]
Other family member	25.8 (21.3–30.9) [191]	15.4 (10.8–21.4) [61]	23.6 (12.8–39.4) [26]	38.5 (30.1–47.5) [89]	16.5 (8.1–30.6) [15]
Spouse or other intimate partner	1.9 (1.0-3.6) [18]	0.4 (0.1–1.1) [4]	6.4 (2.1–17.6) [4]	2.4 (1.1–5.3) [9]	0.1 (0.0–1.0) [1]
Parent/stepparent	3.4 (2.0-5.6) [33]	3.1 (1.6-6.0) [11]	5.8 (2.0-15.7) [7]	3.5 (1.3–8.6) [14]	1.2 (0.2–8.1) [1]
Child/stepchild	0.9 (0.4–1.9) [12]	0.6 (0.2–1.6) [4]	4.0 (1.2–12.0) [4]	0.5 (0.2–1.4) [4]	0
Sibling/stepsibling	5.7 (3.7–8.7) [42]	5.3 (2.6–10.6) [12]	5.6 (2.7–11.4) [10]	5.4 (2.7–10.6) [16]	8.5 (2.2–28.0) [4]
Neighbor	6.5 (4.1–10.2) [45]	8.0 (3.7–16.5) [21]	2.3 (0.5–10.5) [3]	6.2 (3.1–12.0) [18]	7.7 (2.2–23.9) [3]
Someone else	20.0 (16.1–24.5) [164]	29.5 (22.5–37.7) [96]	14.5 (7.2–27.0) [17]	15.5 (10.2–23.0) [43]	10.9 (4.4–24.5) [8]

Numbers in the table are presented as weighted percentages (95% confidence intervals) [unweighted n]

*17 respondents (0.7%) did not answer this question

IQR = interquartile range

**Fig. 1:**
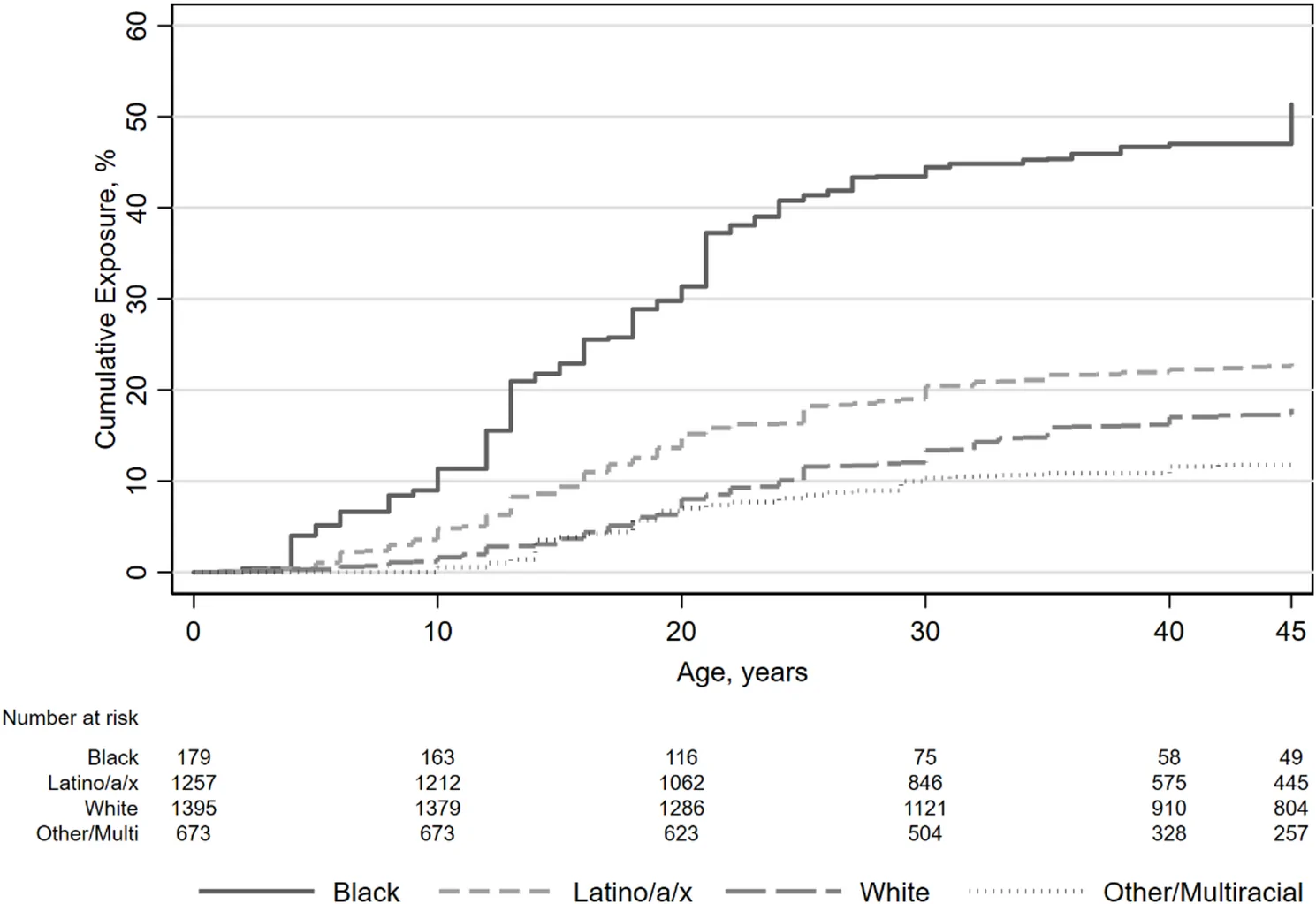
Cumulative exposure to socially proximate firearm violence across the life course, by race and ethnicity, 2024 California Safety and Wellbeing Survey

Figure 1 presents cumulative exposure to socially proximate firearm violence across the life course by race and ethnicity. By age 45, 51.3% (95% CI = 40.9–63.2) of Black individuals and 22.9% (95% CI = 19.2–26.6) of Latino/a/x individuals knew someone who had been shot, compared with 17.9% (95% CI = 15.1–20.8) of White individuals and 12.1% (95% CI = 7.9–16.2) of individuals categorized as other/multiracial. Among Black and Latino/a/x individuals, first exposure often occurred during late childhood and early adolescence, with cumulative exposure increasing sharply during this period. By age 15, 22.9% (95% CI = 12.4–33.4) of Black individuals and 9.4% (95% CI = 6.8–12.0) of Latino/a/x individuals had already known someone who had been shot, compared with 3.7% (95% CI = 2.4–4.9) of White individuals and 3.8% (95% CI = 1.2–6.5) of individuals categorized as other/multiracial.

**Fig. 2:**
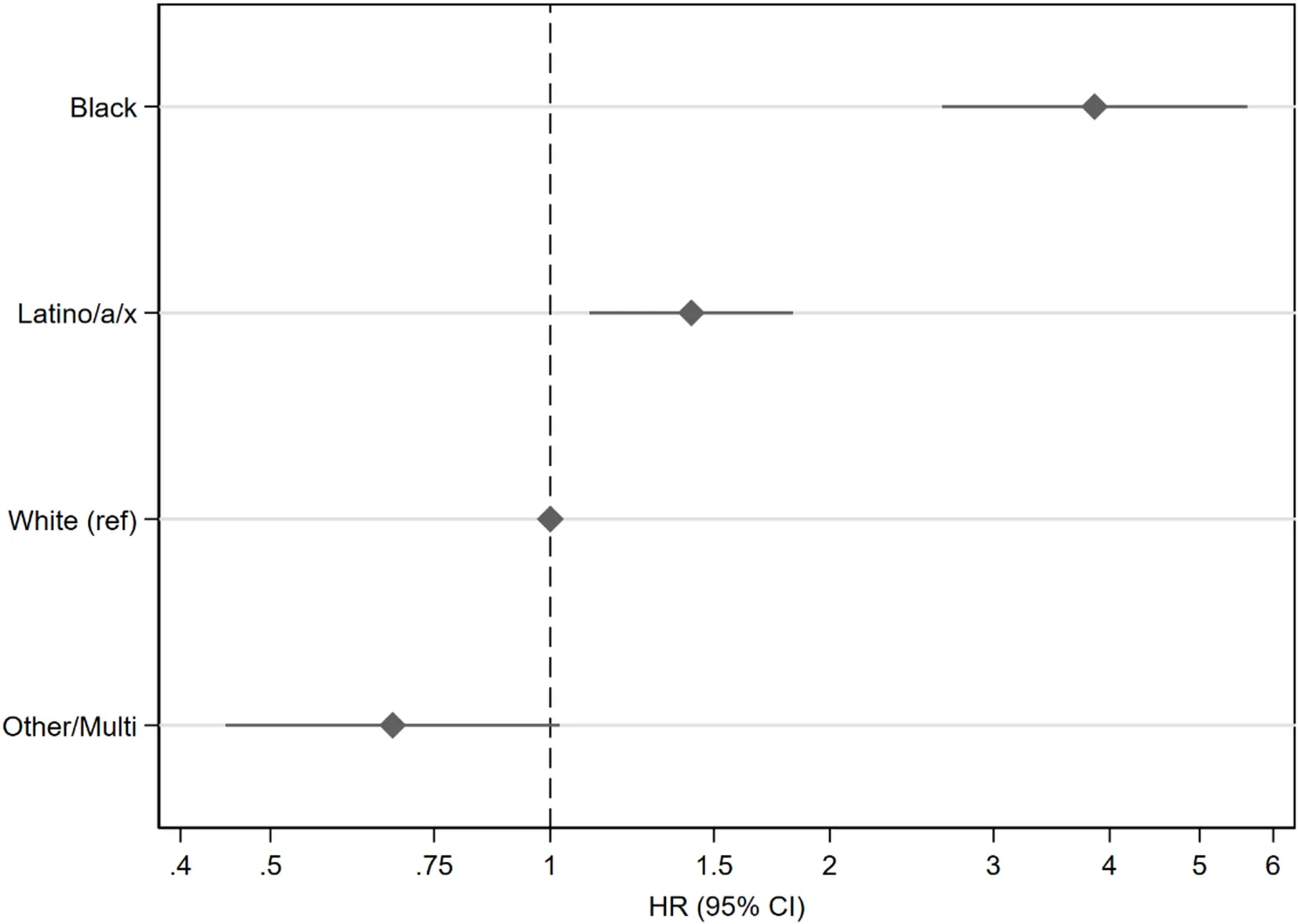
Hazard ratios for socially proximate firearm violence exposure, by race and ethnicity, 2024 California Safety and Wellbeing Survey

Cox proportional hazards models provided formal estimates of exposure inequities (Fig. 2). Black individuals experienced a nearly fourfold higher hazard of knowing someone who had been shot than White individuals (HR = 3.85, 95% CI = 2.64–5.62), while Latino/a/x individuals also exhibited elevated hazards (HR = 1.42, 95% CI = 1.10–1.83). Hazard estimates for other/multiracial individuals did not differ significantly from those for White individuals.

## Discussion

In this statewide survey of California adults, approximately one in five individuals, representing an estimated 6.3 million people, reported ever knowing someone who had been intentionally shot. Exposure was often concentrated, with more than half of exposed individuals reporting that they knew multiple people who had been shot. Consistent with a “linked lives” perspective [19, 20], the findings suggest that the reach and impacts of firearm violence extend beyond direct victims and reverberate through interconnected social relationships and networks that shape lives across the life course.

The findings also underscore marked racial and ethnic inequities, reflecting broader structural and exclusionary forces that create the conditions for firearm violence and pattern the distribution of protective resources. By midlife, more than half of Black individuals and nearly one in four Latino/a/x individuals in California had known someone who had been shot, compared with fewer than one in five White individuals. Particularly striking were disparities during childhood and adolescence. By age 15, nearly one in four Black individuals and nearly one in ten Latino/a/x individuals had already experienced socially proximate firearm violence, compared with fewer than one in twenty White individuals.

The developmental timing of exposure may have important implications for health and wellbeing, particularly because many individuals first encountered firearm violence during adolescence, when traumatic stressors may have heightened influence on emotional development, perceptions of safety, educational engagement, and long-term mental and physical health [17, 18]. Similar patterns of earlier and more rapid accumulation of exposure among younger birth cohorts (Appendix Figure S1) suggest that these experiences may be occurring progressively earlier across successive generations, although further research is needed to disentangle age, period, and cohort effects. Relational context is another important dimension. The first person known to have been shot was most often described as a friend or extended family member, while children, parents, and spouses featured more prominently in the experiences of Black and Latino/a/x individuals.

In terms of policy and practice, the concentration of socially proximate exposure during childhood and adolescence highlights the importance of developmentally appropriate strategies responsive to the lived experiences and priorities of young people. Such approaches may include family- and school-based supports, hospital- and community-based violence intervention programs, trauma recovery services, and other efforts that strengthen the capacity of peers, family, and trusted community members to promote positive development. Investments in culturally responsive, community-rooted systems of support that foster communalistic coping and connectedness may be particularly important for diverse and disproportionately impacted populations [21, 22, 23]. Yet recent reductions in funding for violence prevention, trauma recovery, and basic needs supports risk undermining the very systems, resources, and relationships that promote safety, stability, and healing.

### Limitations

This study has several limitations. First, data were self-reported and therefore subject to recall error. Second, the measure of socially proximate firearm violence exposure did not capture the severity or circumstances of the shooting. Future research should examine how the timing, frequency, and relational proximity of exposure influence health and social outcomes. Third, estimates may underestimate lifetime exposure because individuals at greatest risk of firearm violence may have died before survey participation. Finally, small sample sizes required combining several racial and ethnic groups, potentially obscuring important heterogeneity. Nonetheless, this study provides novel evidence regarding the prevalence, timing, and unequal distribution of socially proximate firearm violence exposure across the life course.

## Conclusion

Exposure to socially proximate firearm violence is widespread among California adults and is associated with substantial racial and ethnic inequities in cumulative burden, age at first exposure, and relational proximity. Recognizing the population health and health equity implications of these unequal patterns of exposure may help inform investments in community-centered violence prevention, trauma recovery, and healing-centered supports that are culturally and developmentally responsive, broadly accessible, and attentive to the structural conditions that shape both violence and wellbeing.

## Supplementary Information

Below is the link to the electronic supplementary material.

Supplementary Material 1.

## Data Availability

The data that support the findings of this study are not yet publicly available because they are part of an ongoing research program. Data will be made publicly available at a later date. Until that time, researchers interested in accessing the data for scientific purposes should contact the corresponding author to discuss data availability and access.
