# Supplementary material for: Socially proximate firearm violence across the life course: a population-based study of California adults
Source: Inj Epidemiol. 2026 Aug 11;13:72. doi: 10.1186/s40621-026-00704-2 (PMC13741631; doi:10.1186/s40621-026-00704-2)

Socially proximate firearm violence across the life course: a population-based study of California adults

Nicole Kravitz-Wirtz and Amanda J. Aubel

Supplementary Information

Appendix Figure S1. Cumulative exposure to socially proximate firearm violence across the life course by birth cohort, 2024 California Safety and Wellbeing Survey

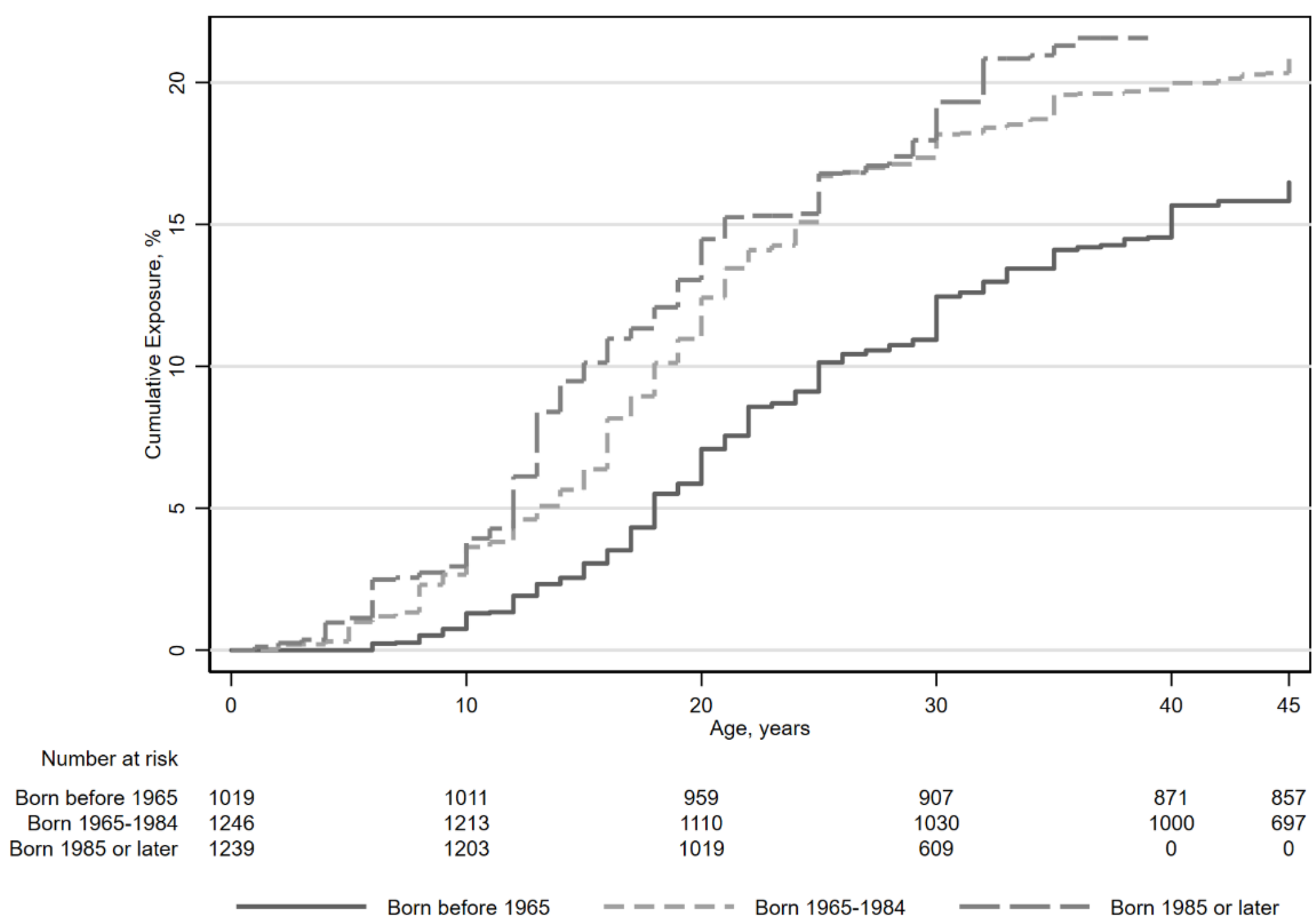

Supplement: Supplementary file 1 [file 40621_2026_704_MOESM1_ESM.pdf]
